# Micro-Scale Genomic DNA Copy Number Aberrations as Another Means of Mutagenesis in Breast Cancer

**DOI:** 10.1371/journal.pone.0051719

**Published:** 2012-12-17

**Authors:** Hann-Hsiang Chao, Xiaping He, Joel S. Parker, Wei Zhao, Charles M. Perou

**Affiliations:** 1 Curriculum in Genetics and Molecular Biology, University of North Carolina at Chapel Hill, Chapel Hill, North Carolina, United States of America; 2 Lineberger Comprehensive Cancer Center, University of North Carolina at Chapel Hill, Chapel Hill, North Carolina, United States of America; 3 Department of Genetics, University of North Carolina at Chapel Hill, Chapel Hill, North Carolina, United States of America; 4 Curriculum in Bioinformatics and Computational Biology, University of North Carolina at Chapel Hill, Chapel Hill, North Carolina, United States of America; 5 The Carolina Genome Sciences Center, University of North Carolina at Chapel Hill, Chapel Hill, North Carolina, United States of America; 6 Department of Pathology and Laboratory Medicine, University of North Carolina at Chapel Hill, Chapel Hill, North Carolina, United States of America; University of North Carolina School of Medicine, United States of America

## Abstract

**Introduction:**

In breast cancer, the basal-like subtype has high levels of genomic instability relative to other breast cancer subtypes with many basal-like-specific regions of aberration. There is evidence that this genomic instability extends to smaller scale genomic aberrations, as shown by a previously described micro-deletion event in the *PTEN* gene in the Basal-like SUM149 breast cancer cell line.

**Methods:**

We sought to identify if small regions of genomic DNA copy number changes exist by using a high density, gene-centric Comparative Genomic Hybridizations (CGH) array on cell lines and primary tumors. A custom tiling array for CGH (244,000 probes, 200 bp tiling resolution) was created to identify small regions of genomic change, which was focused on previously identified basal-like-specific, and general cancer genes. Tumor genomic DNA from 94 patients and 2 breast cancer cell lines was labeled and hybridized to these arrays. Aberrations were called using SWITCHdna and the smallest 25% of SWITCHdna-defined genomic segments were called micro-aberrations (<64 contiguous probes, ∼ 15 kb).

**Results:**

Our data showed that primary tumor breast cancer genomes frequently contained many small-scale copy number gains and losses, termed micro-aberrations, most of which are undetectable using typical-density genome-wide aCGH arrays. The basal-like subtype exhibited the highest incidence of these events. These micro-aberrations sometimes altered expression of the involved gene. We confirmed the presence of the *PTEN* micro-amplification in SUM149 and by mRNA-seq showed that this resulted in loss of expression of all exons downstream of this event. Micro-aberrations disproportionately affected the 5′ regions of the affected genes, including the promoter region, and high frequency of micro-aberrations was associated with poor survival.

**Conclusion:**

Using a high-probe-density, gene-centric aCGH microarray, we present evidence of small-scale genomic aberrations that can contribute to gene inactivation. These events may contribute to tumor formation through mechanisms not detected using conventional DNA copy number analyses.

## Introduction

A hallmark of many human cancers is genomic instability, and cancer itself can be thought of as the result of an altered ploidy [1]. In order to gain a greater understanding of the causes underlying tumor formation, one must understand the core events and changes of cancerous cells. The genetic identity of each cell determines the fate of the cell and thus the cancer genome is a source of information to be mined in order to identify both the ways cancers arise and how they can be treated. The importance of studying the cancer genome cannot be understated as genomic alterations have been linked to cancer causation both broadly and in specific subgroups of patients [2], [3], [4], [5], [6]. Dysregulation of gene expression is one mechanism by which cells become tumorigenic. It has been shown that alterations on a genomic DNA level are likely to cause associated changes in gene expression [7], [8]. Previous global gene expression profiling studies of breast carcinomas have identified at least five distinct subtypes of breast cancer [9], [10], [11], [12], [13] with specific patterns of Copy Number Aberrations (CNA) that can also define genetic events associated with different expression subtypes [14], [15]. The interplay between genomic DNA changes and gene expression is something that can yield much information about the underlying processes that contribute to breast cancer formation and development. Continued investigation of copy number abnormalities in breast cancer is likely to yield additional insights into the pathogenesis of the disease.

As knowledge about breast and other cancers advances, we are finding that there are numerous complex ways by which the genome can be disrupted. While certainly gross chromosomal aberrations and rearrangements have been seen to initiate disease [3], more subtle derangements of the genome can also contribute to tumor formation as well. With the continued advancement in technologies to detect DNA copy number changes, previously difficult to detect varieties of genetic abnormalities are continuing to be discovered. Through the use of a high-density array comparative genomic hybridization (HD-aCGH) platform, it is possible to detect both gross and fine-scale aberrations in genes [16], [17]. Small-scale CNA, here termed micro-aberrations, represent a previously under-investigated source of copy number variation that may shed light on breast subtype characteristics and tumorigenesis. These micro-aberrations have not previously been the focus of any dedicated study and thus we sought to design assays to identify and characterize them. Enhanced detection, cataloguing, and validation of these events could be an avenue through which we can gain a greater understanding of breast cancer genomes through the improved ability to detect genetic events affecting gene expression and function. We focused our investigation on 128 genes shown to be of importance in breast and other cancers; we reasoned that because these genes were frequently disrupted in cancer, they might be more likely to harbor detectable micro-aberrations. Furthermore, any micro-aberrations that might be detected within these genes would be more likely to be biologically relevant and functional than ones that might be detected within a randomly selected panel of genes. Thus, in this study, we utilize a fine-resolution platform to identify these small scale events and examine the functional consequences that result when they are present.

## Methods

### Ethics Statement

All samples used in this study were collected using IRB-approved protocols and all patients signed informed consent forms and the data were analyzed anonymously.

### Breast Cancer Patient Dataset

The dataset used here contained both gene expression and high-density array comparative genomic hybridization (HD-aCGH) copy number data from a set of breast tumors from UNC “HD-UNC94” (n = 94). Additionally, the SUM102 and SUM149 breast cancer cell lines [18] were obtained from Dr. Steve Ethier, and assayed using these high-density tiling arrays. Tumors in the dataset were assayed for gene expression patterns using Agilent DNA microarrays as previously described [19]. Log_2_ ratio data were taken from the UNC Microarray Database (UMD), filtering for a lowess normalized intensity value of 10 or above for each channel, and 70% present data values, and then used for further analyses. Data is available from Gene Expression Omnibus under GSE36889 Sample information including clinical data, subtype, source, GEO Sample ID and overlap with copy number information, can be found in Table S1.

### Classifying Tumors for Gene Expression-based Subtype Classification

The Lowess normalized gene expression R/G Log_2_ ratio data from the HD-UNC94 data set used different gene expression microarray platforms. The dataset was therefore then limited to the probes/genes shared across both platforms. After column standardization of both platforms (samples at N(0,1)), Distance Weighted Discrimination (DWD) [20] was used to remove platform bias prior to classification for the gene expression arrays. After normalization, the R/G Log_2_ ratio data was collapsed (via averaging) from probes to HGNC gene symbols. The PAM50 gene set predictor [19] was used to assign subtypes to the tumors.

### Tiling Array Design

The custom HD-aCGH tiling platform was designed using Agilent’s E-array v5.0 online (https://earray.chem.agilent.com/earray/) software and built on the Human 244 k Custom Oligo platform (GPL15359 Agilent UNC Perou Lab 1X244 k Custom Tiling CGH Array). 230,606 probes cover a total region of 45 Mb, which includes the full genomic sequence of the 128 genes of interest as well as the region 150 kb upstream and downstream of each of these genes (Table S2); this design gave an average resolution of 200 bp between contiguous probes. Labeling and hybridization were performed according to manufacturer’s instructions using the Agilent Genomic DNA Labeling Kit PLUS (Catalogue Number 5188–5309). A Human Genomic DNA Pool (Promega, Catalogue Number G3041) was used as reference DNA, which was compared versus every tumor or cell line sample. Microarrays were scanned on an Agilent DNA Microarray scanner (G2565CA) and the data uploaded to the University of North Carolina Microarray Database (UMD, www.genome.unc.edu).

### Identification of CNA and microCNA with SWITCHdna

To determine regions of Copy Number Aberration (CNA), we utilized the SWITCHdna algorithm [14], focusing on individual genes. For the purposes of this study, the analysis window was limited to the genomic region of each gene and its introns, plus the 5 kb upstream of the start codon, and downstream of the end of the 3′UTR (Table S2). In order to further filter the identified segments, we set the cutoff for the absolute value of the log_2_ ratio to be greater than 0.30 in order to reduce false positives. After identifying all genomic segments of alteration using SWITCHdna, we analyzed the distribution of sizes of aberrant segments and established a cutoff of <64 contiguous SWITCHdna probes, or ∼ ≤15 kb as the definition of a micro-aberration, which equates to the smallest 25% of CNA in this dataset.

In order to identify the regions of genes most commonly affected by micro-aberrations, each gene was divided into four quadrants based upon a proportional splitting of each gene into four equal segments: 5′ End (typically being the promoter region, 5′UTR, beginning regions of gene), 5′ Middle (first ½ of gene), 3′ Middle (second ½ of gene), 3′ End (typically being the end regions of gene, 3′ UTR, downstream region). For every micro-aberration instance, the affected quadrants were tallied for each quadrant, and the proportion of affected quadrants out of all possible quadrants was calculated. Similarly, each micro-aberration instance was assessed in terms of whether it encompassed the promoter or 5′ untranslated region (UTR) of each gene, with the promoter region defined as genomic space upstream of the transcription start site.

### mRNA-seq

mRNA-seq was performed on total RNA isolated from cell lines and tumors using the Qiagen RNeasy Mini Kit (Cat. No. 74104). Library preparation was performed using the TruSeq RNA Sample Kit from Illumina (Cat. No. RS-930-2001) following the low input protocol detailed in the manufacturer’s guidelines. 1×76 bp nucleotide reads were generated using an Illumina GAII sequencer for the SUM102 cell line. For the two tumor samples (UNC990141B and UNC040182B), we sequenced using a 2×50 bp configuration using an Illumina HiSeq2000. In all cases, the read data were aligned to the human HG19 reference genome from the UCSC genome browser [21] **,** and mapped using MapSplice [22]. The alignments were visualized using the Integrative Genomics Viewer (IGV) [23] for evidence of micro-aberrations. To investigate the molecular mechanisms of these aberrations, we next collected the reads aligning to the target regions and performed *de novo* assembly with the Trinity [24]. Default assembly settings were used, and the *de novo* assembled contigs then compared to the reference using BLAST.

### Survival Analysis

The patients in the dataset were rank ordered by total number of SWITCHdna-defined aberrations, and micro-aberrations, and separated in the top 67% and bottom 33%; additional rank order splits were also evaluated, but data not shown. Survival analyses were performed using the Kaplan-Meier test in R [25].

## Results

### Copy Number Micro-aberrations are Present in Breast Tumor Subtypes

**Figure 1:**
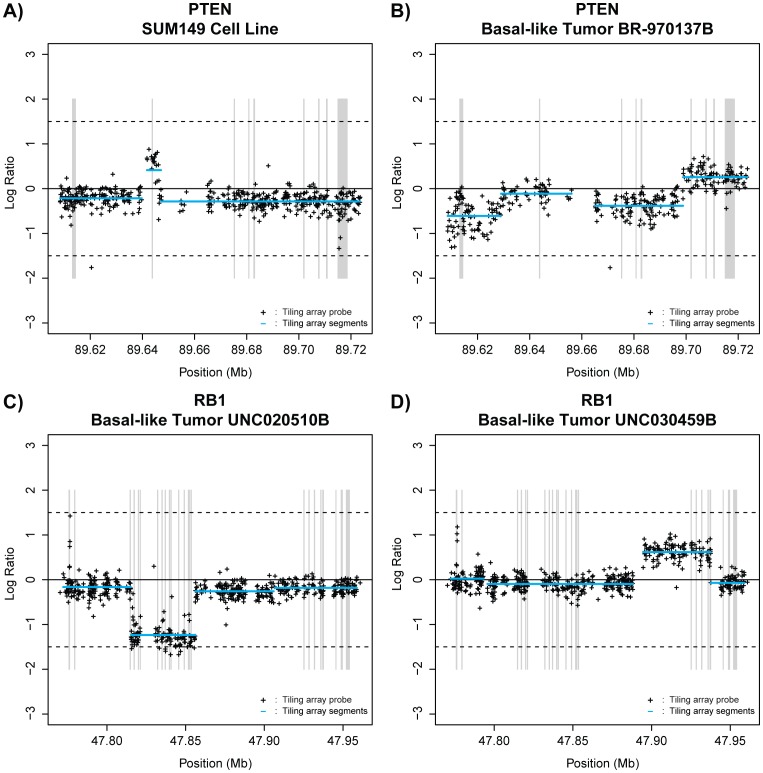
Selected examples of intra-genic micro-aberrations. A) The previously identified *PTEN* exon 2 micro-amplification in SUM149 cell line DNA. B) Intra-genic deletion in *PTEN* in basal-like tumor BR-970137B. C) Focal deletion of *RB1* in basal-like tumor UNC020510B, and D) amplification of *RB1* gene in basal-like tumor UNC030459B. The locations of the tiling array probes are indicated with black crosses. Exons are highlighted with grey bars. SWITCHdna called segments are indicated in blue.

In order to test the hypothesis that primary breast cancer genomes contain areas of small-scale copy number gains and losses, termed micro-aberrations, we designed a custom, high-resolution, high-density, comparative genomic hybridization tiling array (HD-aCGH) with an average probe spacing of 200 base pairs, and which was focused on 128 selected genes (Table S2). We assembled a dataset of 94 tumors and 2 cell lines and tested them on this HD-aCGH array. Each tumor was also classified into one of five previously defined gene expression subtypes using the published PAM50 identifier [19], and genomic DNA copy number aberrations identified using the SWITCHdna algorithm [14]. Using this HD-aCGH array, we were able to identify both previously observed large scale amplifications and deletions, and novel small-scale copy number aberrations, which we have highlighted a few selected examples here; the SUM149 cell line has a previously identified micro-amplification in exon 2 of the PTEN gene [16], which we also clearly observed using our HD-aCGH platform (Fig. 1A). A number of other relatively small intra-genic aberrations were also detected, including a focal deletion in PTEN in a basal-like tumor (Fig. 1B), an intra-genic deletion in RB1 in a different basal-like tumor (Fig. 1C), and a small RB1 amplification in yet a third basal-like tumor (Fig. 1D).

To objectively define a micro-aberration, we established a size definition of the smallest 25% of SWITCHdna identified aberrations in this dataset, which resulted in the size cut off of approximately 64 contiguous probes (∼15 kb). The previously identified PTEN micro-amplification in the SUM149 cell line is able to be identified using these criteria. The genomic landscape of the basal-like subtype exhibited many of these micro-aberrations, as basal-like tumors showed the highest incidence of these events (Table 1).

**Table 1:** Copy Number Micro-aberrations by Subtype.

	Average Micro-aberrations/Sample	Median Micro-aberrations/Sample	% Samples with Micro-aberration
Subtype			
Basal-like (n = 31)	4.29	4	83.87
Luminal A (n = 27)	3.07	1	74.07
Luminal B (n = 21)	3.81	2	80.95
HER2-enriched (n = 10)	2.90	2	80.00
Normal-like (n = 5)	2.00	0	40.00

The mean and median numbers of micro-aberrations for samples within each subtype are shown, as is the percentage of samples within each subtype that exhibited any micro-aberrations.

**Figure 2:**
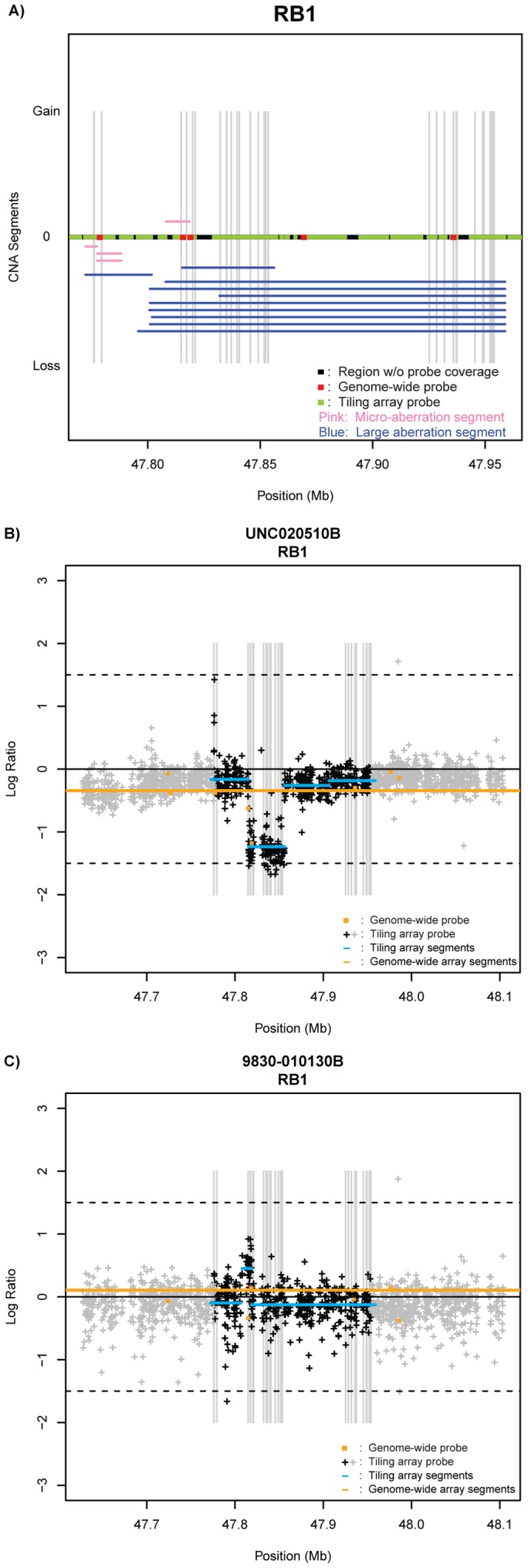
Comparison of HD-aCGH tiling array and 109K Illumina SNP platform generated SWITCHdna copy number segments for RB1. A) Using the 49 samples in common between these two platforms, each copy number aberrant segment called by SWITCHdna is plotted by its location within the *RB1* gene. Pink segments indicate micro-aberrations and blue segments indicate macro-aberrations. Exons are highlighted in grey bars. The locations of the tiling array probes are indicated with green segments and the corresponding locations of probes from a previously used 109K Illumina genome-wide SNP platform are indicated in red**.** Regions without any probe coverage are colored black. B, C) Two representative examples are shown illustrating the different detection thresholds achieved by the tiling array platform and the 109 k SNP platform for CNA in *RB1*. Exons are highlighted in grey bars. The locations of the tiling array probes are indicated with crosses, where black crosses represent probes within the gene and grey crosses indicate probes in the upstream/downstream regions of the gene. The corresponding locations of the 109K genome-wide SNP probes are indicated with orange circles**.** SWITCHdna called segments for each platform are also shown with tiling array segments in blue and 109K platform segments in orange.

The value of the high-density tiling array platform is also shown in another example of a RB1 alteration (Figure 2). Each identified micro- and macro-aberration segment for this gene is plotted with the RB1 exons identified by each gray stripe, along with the location of each RB1 probe on the tiling array (green segments), and also shown are probes from an earlier 109,000 feature single nucleotide polymorphism (SNP) platform [14], which was used in a previous study examining whole-genome landscapes of breast tumor subtypes (5 probes: red lines) (Figure 2A). The micro-aberration segments overlap with at most one genome-wide probe from the 109K arrays, and thus would have never been called a loss given how most aCGH programs call “changed segments”, which was the case for SWITCHdna. In addition, several macro-aberrations identified from the tiling array platform have minimal overlap with the 109K genome-wide probes. 49/94 samples assayed here on the HD-aCGH array had previously been assayed on the 109K SNP platform and the results of this overlap set for RB1 were compared directly in terms of CNA assignment agreement by SWITCHdna. Again, focusing on the RB1 gene as our example case, we observed copy number aberrations in six of these 49 samples by the HD-aCGH array for RB1; only two of these six samples’ CNA were detectable by the 109K SNP array and these were the aberrations that spanned the whole gene. The remaining four, all of which were intra-genic events, were missed by the copy number segments generated from the whole-genome array (data not shown). This is illustrated in two example RB1 gene plots directly comparing the probes and segments from the high-density tiling array and the 109 k SNP array (Figure 2B, 2C).

### Small Scale Copy Number Aberrations can Affect Gene Expression

**Figure 3:**
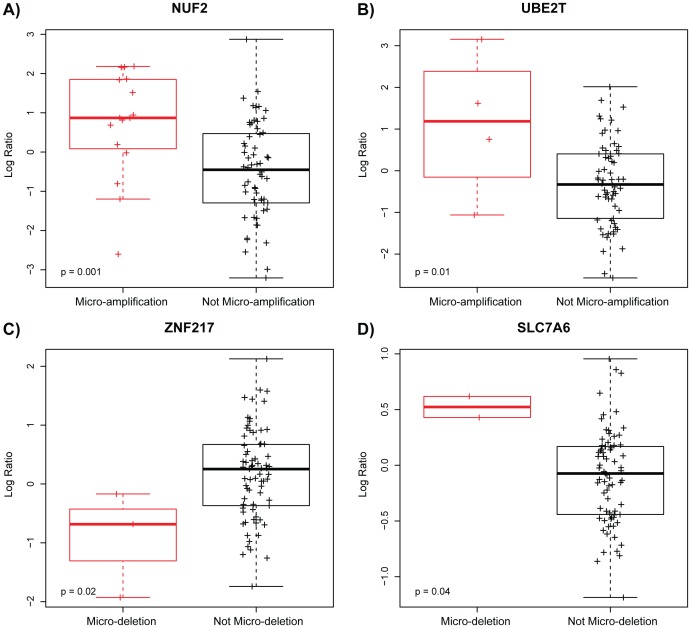
The presence of micro-aberrations can result in differential expression by copy number status. Samples with micro-amplifications in A) *NUF2* and B) *UBE2T* are associated with significantly higher expression of the gene than samples without these aberrations. Samples with micro-deletion in C) *ZNF217* are associated with significantly lower expression of the gene than samples without these aberrations. Samples with micro-deletion in D) *SLC7A6* are associated with significantly higher expression of the gene than samples without these aberrations.

Beyond simply determining the frequency of micro-aberrations present within each gene or tumor, we wanted to assess whether the presence of micro-aberrations would result in functional consequences. We first assigned a copy number status for each gene and each sample (gross copy number gain, gross copy number loss, micro-amplification, or micro-deletion) and then for each gene, determined whether the corresponding gene expression was concordant with the type of genomic aberration observed (i.e. micro-amplifications result in increased expression and/or micro-deletions result in decreased expression of the involved gene). We found similar rates of concordant expression between micro-aberrations and gross aberrations, with 30–40% of the tested genes showing 100% agreement between aberration type and gene expression, meaning that for these genes, every sample that displayed a micro-amplification in the gene also had greater than median expression of the gene and likewise every sample that had a micro-deletion in the gene had less than median expression of the gene. Another 50–75% of the tested genes showed at least 50% concordance between aberration type and gene expression meaning at least half of the samples that displayed a copy number aberration had altered expression of the affected gene in the same direction as the CNA (Table 2). These findings suggest that the micro-aberrations have functional effects upon gene expression similar to what is seen with larger scale CNA. We also examined the expression status of each micro-aberration by micro-aberration type to determine if there was an association between micro-amplifications and high expression and micro-deletions and low expression across all events instead of within genes. When all micro-aberrations are combined, there is no significant difference in expression level between samples with micro-aberrations versus those without, but significant differences by Fisher’s Exact test were observed when looking within micro-amplifications or micro-deletions (Table 3). A number of genes containing micro-aberrations also showed differential expression of the involved gene when comparing the aberrant vs. non-aberrant groups by ANOVA (Figure 3). ANOVA box plots are shown for the genes NUF2 (Fig. 3A) and UBE2T (Fig. 3B), where samples with micro-amplifications had significantly higher expression of the gene than those without micro-amplifications. Also shown is ZNF217 (Fig. 3C), where samples with micro-deletions had lower expression of the gene than those without micro-deletions, and SLC7A6 (Fig. 3D) where the samples with micro-deletions had higher expression than the samples that do not; we do note that the sample size is small in some cases, but overall these data suggest that micro-aberrations can affect gene expression.

**Table 2:** Analysis of Concordant Expression by Aberration Type.

		# of Genes with CNA with Concordant Gene Expression			
		Gross Gains	Gross Losses	Micro-amplifications	Micro-deletions
# 100% Concordant/# with Aberration	(%)	32/105 (30.5%)	25/83 (30.1%)	24/64 (37.5%)	22/53 (41.5%)
# > = 50% Concordant/# with Aberration	(%)	79/105 (75.2%)	54/83 (65.1%)	34/64 (53.1%)	36/53 (67.9%)

Analysis within genes of concordant expression by aberration type. Results are shown for gross copy number gains, gross copy number losses, micro-amplifications, and micro-deletions and the frequency that genes are 100% concordant by gene expression or > = 50% concordant by gene expression. A concordant sample is defined as a sample where a copy number gain, or micro-amplification, is accompanied by high expression of the affected gene or a copy number loss or micro-deletion is accompanied by low expression of the affected gene.

**Table 3:** Analysis of Gene Expression Relative to Median Expression by Aberration Type.

Micro-aberrations by Expression Status			
	Micro-amplifications		
	Micro-aberration	No Micro-aberration	Fisher’s Exact Test p-value
Greater than Median Expression	68	4601	
Less than Median Expression	65	6927	p = 0.01
	**Micro-deletions**		
	**Micro-aberration**	**No Micro-aberration**	**Fisher’s Exact Test p-value**
Greater than Median Expression	54	6898	
Less than Median Expression	61	4914	p = 0.02
	**All Micro-aberrations**		
	**Micro-aberration**	**No Micro-aberration**	**Fisher’s Exact Test p-value**
Greater than Median Expression	122	11499	
Less than Median Expression	126	11841	p = 1

For micro-amplifications, micro-deletions, and all micro-aberrations combined, the number of samples with or without micro-aberration and with greater than median or less than median expression are displayed. P-value calculated by Fisher’s Exact Test.

### Genomic Micro-amplification Causes Exon Skipping

**Figure 4:**
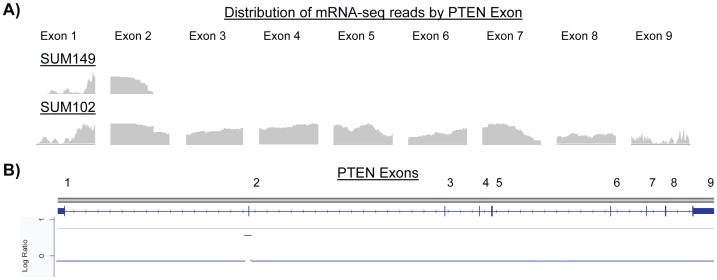
Micro-amplification in *PTEN* in the SUM149 cell line disrupts exon expression. A) The distribution of mRNA-seq reads by exon is shown for the SUM149 and SUM102 cell lines. B) For reference, the genomic space of the *PTEN* gene is shown, along with the copy number status by SWITCHdna across the *PTEN* gene in SUM149.

We performed a closer examination of the micro-amplification of the PTEN gene in the SUM149 cell line, as it is a validated aberration that has now been identified by multiple groups. Using mRNA-seq data, we assessed the expression of the PTEN gene on an exon level to determine the functional consequence of the DNA micro-amplification. For comparison, we also examined the data for the SUM102 cell line, which has no genomic alterations in PTEN (data not shown). The distribution of aligned reads for each exon is shown for each cell line (Figure 4A), with the SWITCHdna copy number segments for SUM149 shown in genomic space for reference (Figure 4B). In SUM149, there is a lack of PTEN gene expression starting from the middle of exon 2, which coincides with the location of the genomic DNA micro-amplification. In comparison, the SUM102 cell line has aligned reads throughout the entirety of the PTEN gene, thus the micro-amplification in SUM149 causes a loss of expression of all downstream exons.

Additionally, we sought to validate other micro-aberrations using mRNA-seq data. We were able to observe instances where the presence of a micro-aberration resulted in production of inter-exon mRNA reads with sequence infidelity. In the 990141B tumor sample, the presence of a micro-amplification in the *EGFR* gene results in many mRNA-seq reads that mapped outside of the exon, and that often contain misalignments (Figure S1). This finding was not observed when examining data from the SUM102 cell line data that does not contain a similar micro-aberration. Similarly, in the UNC040182B tumor sample, a micro-deletion in the *BCL11A* gene results in mRNA-seq reads that align outside of the exon containing multiple sequence errors, a finding likewise not observed in the unaffected SUM102 cell line data (Figure S2).

We pursued further analysis by performing *de novo* assembly of mRNA-seq reads that mapped to the region of *EGFR* micro-amplification in the 990141B tumor sample and generated two contigs that aligned to the reference mRNA sequence, aside from 5 bases at the start of the contig and 3 bases at the end of the contig (Figure S3). The 5 base unaligned sequence occurs twice within the aligned reference region and may represent the site joining a duplication of the stretch of genome that results in the micro-amplification. *de novo* assembly of mRNA-seq reads that mapped to the region of *BCL11A* micro-deletion in the UNC040182B tumor sample generates a contig that begins at the end of the of the affected exon and extends into intronic space (Figure S4); this could be attributed to loss of the beginning of the exon, which then causes mis-splicing and the generation of an altered mRNA. Thus in these two cases, the micro-aberrations affected the mRNA structures.

### The 5′and Promoter Regions of Genes are most Commonly Affected by Micro-aberrations

**Figure 5:**
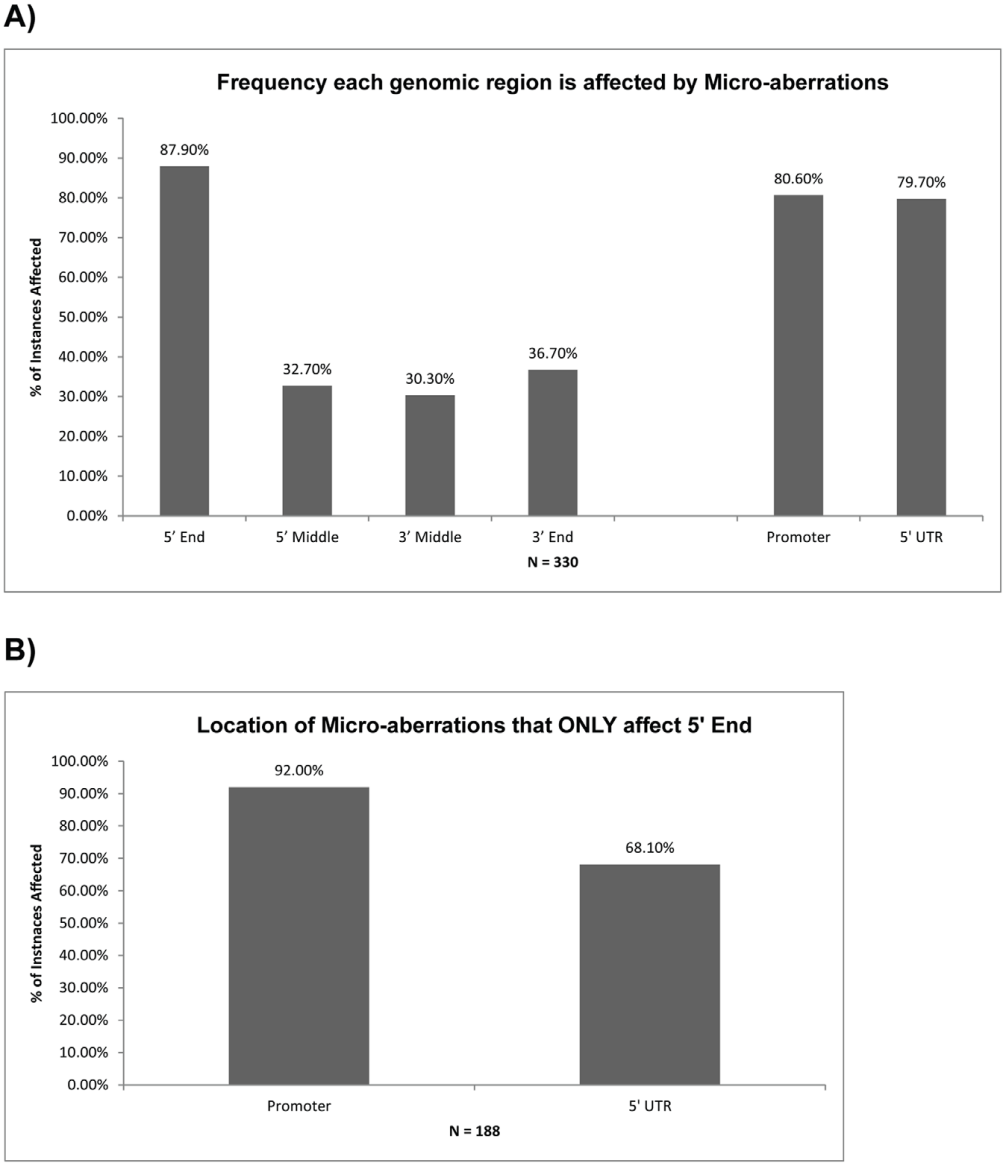
Frequency that genomic quadrants are affected by micro-aberrations. A) The percentage of total possible instances that a given genomic region is overlapped by a micro-aberration segment is displayed for each genomic quadrant (5′ End, 5′ Middle, 3′ Middle, 3′End) and the 5′ UTR and promoter regions. B) For the micro-aberrations that only affected the 5′ End region, the % of instances where it affected the promoter or 5′ UTR is listed.

In order to assess whether certain regions of genes were more commonly affected by micro-aberrations than others, we portioned each of the 128 genes on the HD-aCGH array into four quadrants: 5′ End, 5′ Middle, 3′ Middle, and 3′ End, based upon a proportional splitting of each gene into 4 equal segments. For every micro-aberration instance (n = 330), we noted the quadrants that it occupied, and then for each quadrant, determined what proportion of the total possible quadrants were affected by a micro-aberration event (Figure 5A). We found that the 5′ end of the gene was disproportionately affected by micro-aberrations (88% vs. <40%). Additional refinement of the affected region was also performed and a large percentage of micro-aberrations also affected the promoter (defined as the area upstream of the coding region) and 5′ UTR regions as well (80.6% and 79.7% respectively). Of the aberrations whose area of effect was limited only to the 5′ End, the promoter region was affected at a higher rate than the 5′ UTR region (Figure 5B, 92.0% vs. 68.1%).

### Micro-aberration Frequency Associated with Poorer Survival

**Figure 6:**
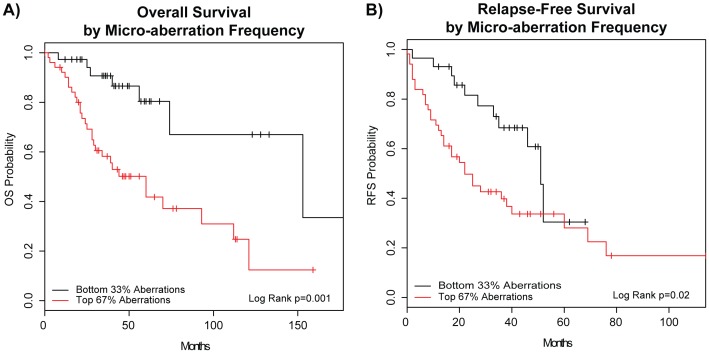
Higher levels of micro-aberrations are associated with worse survival outcomes. A) Kaplan-Meier plots for overall survival and B) relapse-free survival are shown for the patients in the tiling array datasets. The patients were split into two groups, the top 67% in terms of total micro-aberration versus the bottom 33%. (N = 94).

The survival outcomes of patients with varying levels of copy number aberrations were also assessed to determine if an association was present. We identified the number of copy number micro-aberrations per sample using our SWITCHdna criteria and rank-ordered the patients in terms of micro-aberration frequency. Each patient in the HD-UNC 94 dataset was assigned to one of two groups depending on whether they were in the top 67% of microCNA or the bottom 33%. Kaplan-Meier analysis was performed examining overall (Fig. 6A) and relapse-free survival (Fig. 6B). We saw that patients with the least genomic instability as assessed by SWITCHdna-called micro-aberrations had significantly better outcomes in terms of both overall and relapse-free survival. A caveat to these analyses is that the sample size is small, and additional rank order splits of the data were only trending towards significance, but similar to what has been seen before for large numbers of large scale changes [26], [27], tumors with the most numbers of changes tended to show worse survival.

Lastly, we examined the frequency of micro-aberrations for each of the 128 genes tested (Table S3). Here, we list the top 17 most micro-aberrant genes among those that were tested on this tiling array with breakdown by micro-amplification and micro-deletion (Table 4). We also show the number of micro-aberrations that would be expected by chance for each gene based on the distribution of log-ratio values within our dataset and our cutoffs for micro-aberrations. Genes such as *MYC* and *PIK3CA,* known to be activated in many cancers tend to show more micro-amplifications; others known to be inactivated in cancer such as *RB1* and *PTEN* display comparatively more micro-deletions. Gene Set Enrichment Analysis was performed using DAVID [28] on the genes exhibiting more than one micro-aberrations in our study, where the background for the analysis was limited to the 128 genes present on the tiling array in order to control for our biased initial selection of genes. We observed that micro-aberrant genes were more likely to be involved in interphase of the mitotic cell cycle (Table 5).

**Table 4:** Micro-aberration Frequency by Gene.

Top 17 Genes with the Most Micro-aberrations				
	Gene Name	# of Micro-aberration Occurrences(All/Amp/Del)	# Micro-aberrations Expected by Chance*	p-value**
1.	NUF2	23/22/1	0	<.001
2.	NAT1	21/0/21	0	<.001
3.	FZD7	11/4/7	0	<.001
4.	MYC	11/11/0	0	<.001
5.	ELOVL5	11/5/6	0	<.001
6.	PIK3CA	11/8/3	0	<.001
7.	CENPF	10/9/1	0	0.002
8.	UBE2T	9/7/2	0	0.003
9.	S100A11	8/7/1	0	0.007
10.	ZNF217	8/5/3	0	0.007
11.	RB1	7/2/5	0	0.01
12.	CCNB1	7/0/7	0	0.01
13.	CELSR1	6/3/3	0	0.03
14.	MIA	6/4/2	0	0.03
15.	TP53BP2	5/4/1	0	0.06
16.	MDM2	5/4/1	0	0.06
17.	PTEN	5/2/3	0	0.06

A) The top 17 genes that displayed the most micro-aberrations are shown, along with the number of micro-aberrations (All)/micro-amplifications (Amp)/micro-deletions (Del) seen within each gene. The expected number of micro-aberrations for each gene, corrected for gene size, is calculated based on the distribution of log_2_ ratio values for our dataset and the probability of observing a segment meeting our micro-aberration cutoffs. p-values calculated by Chi-square test.

* Based on the distribution of log-ratio data within our dataset and the probability given this distribution of contiguous probes meeting the cutoffs for micro-aberrations, with correction for gene size.

** p-value based on Chi-square test.

**Table 5:** Gene Set Enrichment Analysis of Micro-aberrant Genes.

Gene Set Name	# Genes in Overlap	Fisher Exact p-value
GOTERM_BP_FAT: Interphase of Mitotic Cell Cycle	13	.034

Results of a Gene Set Enrichment Analysis of the genes exhibiting more than one micro-aberration are displayed, showing an enrichment of a cell-cycle related pathway.

## Discussion

The previous discovery of micro-aberrations within genes [16] using a high-density aCGH array, and the lack of description of such features in many whole-genome aCGH-based breast cancer studies [14], [15], [29], [30] suggests that these micro-aberrations may occur regularly in breast cancer genomes and that they simply have not yet been detected in previous studies due to the resolution of typical SNP-based aCGH platforms. To address this hypothesis, we assembled a dataset of 94 breast tumors and two breast cancer cell lines and tested them on a custom-designed aCGH tiling array; this array was targeted to 128 gene panel focused on important cancer relevant genes, and previously identified basal-like cancer specific regions and genes (Table S2) [14], [15], [31], [32].

By utilizing a previously tested segmentation and aberration calling algorithm called SWITCHdna [14], we analyzed the tiling array data and proceeded to generate a numerical definition of a micro-aberration (<64 probes, ∼15 kb). Essentially all of these micro-aberrations would be mostly undetectable using lower-resolution genome-wide platforms, as these segments would be covered by at most one probe on such arrays (Figure 2). An analysis of the frequency of micro-aberrations within our dataset samples suggests that the basal-like subtype had the most frequent occurrences of these events, mirroring their high overall genomic instability [15], [30], [33], [34], [35]; thus the presence of micro-aberrations did correlate with the presence of large aberrations. We note that the frequency of micro-aberrations observed in our dataset was similar to that for both gross copy number aberrations and single-nucleotide variants, when we examine the same genomic regions targeted in this study for comparable distribution of tumor samples (data not shown).

Our data also shows that at least in some cases, these events have functional downstream consequences (Fig. 3 and 4, Table 2 and 3, Fig. S1 and S2). One point of discussion is the mechanisms by which these micro-aberrations might lead to altered gene expression. It is intuitive how micro-deletions might result in decreased gene expression of the affected gene. However, it is less clear how micro-amplifications can result in increased expression of the affected gene. One proposed mechanism is that micro-amplifications might preferentially occur in the 5′ promoter site, given the overall predilection for micro-aberrations to occur in that region, and those micro-amplifications that occur in the promoter have higher rates of positive concordance than those that occur elsewhere. This did not appear to be the case within this dataset (data not shown), but is a mechanism worth considering for expanded studies. Another mechanism could be that the affected gene is disrupted in a heterozygous fashion, and upregulation of the remaining copy occurs as a result. The current platform is not designed to distinguish between homozygous and heterozygous change, but this is a mechanism by which altered expression might occur.

The finding of exon skipping at the point of the focal amplification in the PTEN gene in the SUM149 cell line is particularly interesting given the otherwise normal copy number for this gene in this cell line. The exact mechanism that induces this exon skipping is yet to be determined, but one can imagine that some aspect of the amplified DNA sequence results in an alteration to the pre-processed transcript that could cause early truncation or some sort of structural interference [36], [37]. Likewise, the presence of the micro-aberrations in the 990141B tumor sample with *EGFR,* and the UNC040182B tumor sample and *BCL11A,* may cause mRNA mis-processing because the micro-aberration alters the DNA sequence in such a way that splice-site junctions are altered and we are able to observe inter-exon mRNA-seq reads. Using targeted *de novo* assembly of 990141B mRNA-seq data, we are able to generate contigs that suggest an in-place tandem duplication of a region of *EGFR* that may be the cause of the micro-amplification detected in the tiling array and the source of expression disruption. When examining data on the UNC040182B tumor sample, we are able to produce a contig that covers only the latter portion of the affected exon, but extends out into intronic space. The micro-deletion located in this region may knock out the initial portion of the exon, resulting in the production of the observed aberrant mRNA (Figure S3 and S4).

We also found that the 5′ end of genes tended to be the most heavily affected by micro-aberration events, specifically the promoter region of the gene (Figure 5). It is unclear what leads to this predilection, but it does suggest that this specific portion of the gene may be more prone to this type of genomic alteration. The involvement of the promoter region does suggest that the site of active transcriptional processing may lead to a structural genomic weakness that causes a predisposition towards micro-aberrations. This finding may also aid in explaining either the factors involved in the formation of micro-aberrations versus macro-aberrations or the possible downstream consequences of such events.

A limitation of our study is that we are currently unable to determine if any of these micro-aberrations are subtype specific. If they are, this would 1) mirror whole genome study findings, and 2) potentially showcase an alternative means of gene disruption that unravels previously unexplained expression data. As one example, RB1 dysfunction has been shown to be associated with the basal-like subtype [38]. We have found in our own studies, that RB1-LOH was highly correlated with gene expression subtype and patient outcomes, while RB1 protein expression on the same samples was not [39]. An intra-genic micro-aberration could potentially explain such cases, as the genetic abnormality may only affect a portion of the gene such that a protein is still produced and the majority of it intact, but it does not function properly. Expanded studies with high-resolution platforms like whole genome sequencing will allow us to answer this question with increased precision.

Future studies with whole-genome sequencing technology and data are also an additional avenue by which these micro-aberrations can be validated, detected, and further defined. Varying amounts of sequencing depth would, however, be needed depending on the nature of the micro-aberration. Micro-deletions should be comparatively easier to detect as they are due to the absence of DNA, thus reasonably low coverage should be sufficient to call these events. Micro-amplifications would be more complex, especially if one did not know in advance what the nature of the micro-amplification was. Thus, in order to reliably identify all micro-aberrations, one would need sequencing depth capable of performing a *de novo* whole genome assembly [40], [41]. Targeted assembly is able to yield some insights (Figure S3 and S4), but full assembly would undoubtedly generate more answers.

From a broader viewpoint, it stands to reason that other tumor types may also exhibit these types of events, but as yet they have not been widely described [42], [43]. However, similar intragenic deletions in *RB1* and *PTEN* were recently described in melanoma cell lines (SKMEL-207, A2058, and SKMEL-178 [17]), suggesting that these types of micro-genomic events are present in other cancers.

In examining the specific genes on the tiling array that displayed micro-aberrations, we noted that genes that were involved in interphase of the mitotic cell cycle were particularly prone to these small-scale events (Table 5, Table S3). Given that our panel of targeted genes was focused on cancer relevant genes, there was some inherent enrichment for genes of this Gene Ontology class, but even within the background of genes on the array itself, there was a statistically significant enrichment for cell cycle genes. Coupled with our finding of micro-aberrations being localized to promoter regions, we speculate that cell cycle genes are more prone to these events because of their consistent and often high level of transcriptional activity.

We were also able to make the observation that higher genomic instability in the form of micro-aberrations in our dataset was associated with worse survival outcomes (Figure 6). An overall high level of genomic instability has been found to associate with worse survival [26], [27], and here we see that finding extended to micro-instability in our dataset. There was however, a high concordance of overlap between patients that had many large scale changes and many micro-aberrations, and overall, patients with higher total numbers of CNA were associated with poorer outcomes (data not shown). Nonetheless, the same rank-ordering split that was performed on the micro-aberrations did not result in identical findings for overall aberrations, suggesting that while there could be confounding of the micro-aberration survival findings by overall genomic instability, there may also be characteristics unique to the micro-aberrations themselves. Furthermore, the concordance between gross aberrations and micro-aberrations suggests that there may be common mechanisms of genomic instability at play, which may yield insights into how micro-aberrations arise.

### Conclusions

In addition to exhibiting gross copy number changes, breast tumor genomes contain focal micro-aberrations as well when examined using a high-resolution platform. These micro-aberrations occur within the background of global genomic instability and can have disruptive effects upon gene expression. These micro-events represent a potential means of mutagenesis in genes that have been otherwise determined to be normal in terms of gross copy number or SNV-based somatic mutations. Continued investigation into these events with improved tools will allow their increased detection and likely highlight their importance as an additional means of altering gene function.

## Supporting Information

Figure S1 **mRNA-seq read distribution for** ***EGFR*** **in the 990141B tumor sample.** The distribution and alignment of mRNA-seq data for the 990141B tumor sample (top lane) and SUM102 cell line sample (bottom lane) for the *EGFR* gene is visualized using IGV. The corresponding tiling array copy number plot for the tumor sample and gene and the associated area of genomic coverage is highlighted in the bottom panel.
(PDF)

Figure S2 **mRNA-seq read distribution for** ***BCL11A*** **in the UNC040182B tumor sample.** The distribution and alignment of mRNA-seq data for the UNC040182B tumor sample (top lane) and SUM102 cell line sample (bottom lane) for the *BCL11A* gene is visualized using IGV. The corresponding tiling array copy number plot for the tumor sample and gene and the associated area of genomic coverage is highlighted in the bottom panel.
(PDF)

Figure S3 ***de novo*** **assembly of targeted** ***EGFR*** **micro-amplification mRNA-seq data in the 990141B tumor sample.** A) The two contigs aligned to the region of *EGFR* micro-amplification are visualized in space using the UCSC genome browser. B) The aligned region of the contigs are displayed in red with the unaligned base pairs at the start and end of the sequence written out. Sites within the aligned region where the unaligned GACCT sequence are observed are highlighted with black boxes.
(PDF)

Figure S4 ***de novo*** **assembly of targeted** ***BCL11A*** **micro-deletion mRNA-seq data in the UNC040182B tumor sample.** The contig aligned to the region of *BCL11A* micro-deletion is visualized in space using the UCSC genome browser, with the location of the reference exon site displayed. A magnified view of the region is also provided.
(PDF)

Table S1 Clinical data table. Table containing all information on the human breast samples used in this study including outcomes data, microarray and GEO accession number IDs.
(XLSX)

Table S2 Gene list, tiling array coverage, and analysis windows. List of the 128 genes on the tiling array with EntrezGene ID along with their genomic coordinates by NCBI build 36.1 (hg18) of the reference human genome. Coordinates are also provided showing the total coverage on the tiling array for each gene as well as the +/−5 kb analysis window that was used.
(XLSX)

Table S3 Micro-aberration information table. Table containing information on all micro-aberrations identified using the defined parameters described in this manuscript from the tiling array. Information for each listed micro-aberrant segment includes Sample name, Subtype, Affected gene, Start position, Stop position, and Average log-ratio for the segment.
(XLSX)
