# Supplementary figures and images for: Micro-Scale Genomic DNA Copy Number Aberrations as Another Means of Mutagenesis in Breast Cancer
Source: PLoS One. 2012 Dec 17;7(12):e51719. doi: 10.1371/journal.pone.0051719 (PMC3524128; doi:10.1371/journal.pone.0051719)

Supplementary Figure 1

990141B-244k-customCGH

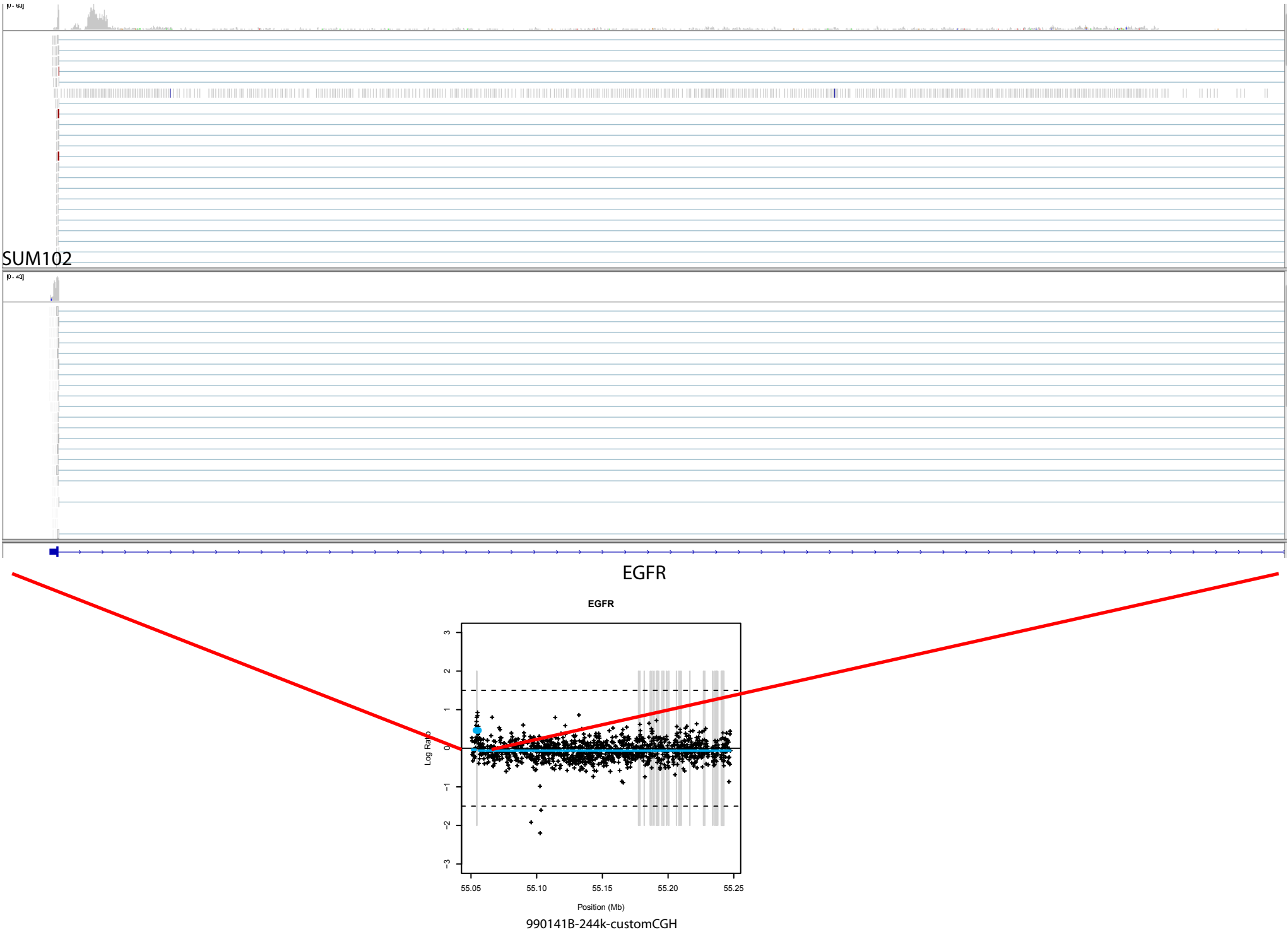

Supplement: Figure S1 — mRNA-seq read distribution for EGFR in the 990141B tumor sample. The distribution and alignment of mRNA-seq data for the 990141B tumor sample (top lane) and SUM102 cell line sample (bottom lane) for the EGFR gene is visualized using IGV. The corresponding tiling array copy number plot for the tumor sample and gene and the associated area of genomic coverage is highlighted in the bottom panel. (PDF) [file pone.0051719.s001.pdf]

Supplementary Figure 2

UNC040182B-244k-customCGH

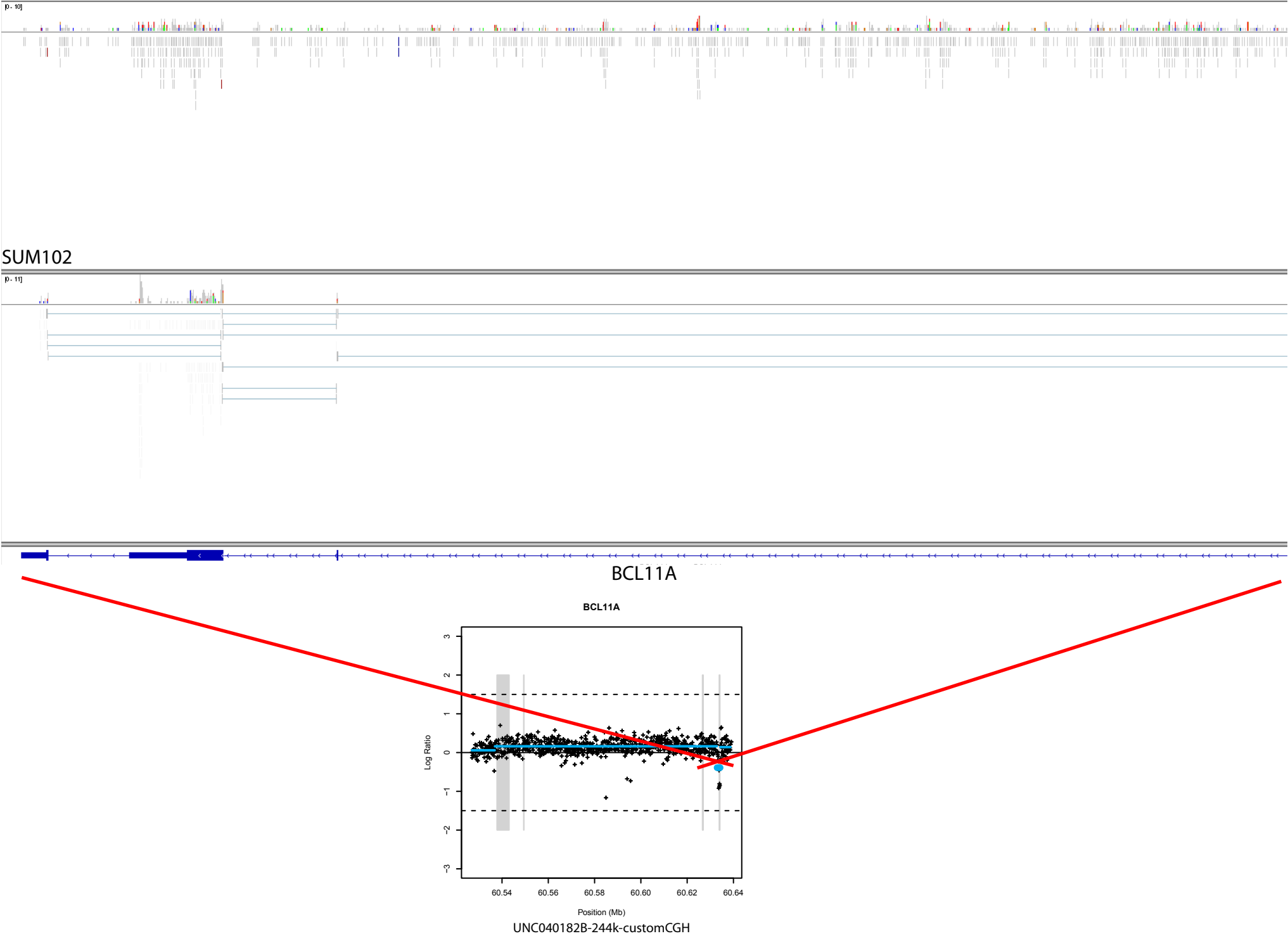

Supplement: Figure S2 — mRNA-seq read distribution for BCL11A in the UNC040182B tumor sample. The distribution and alignment of mRNA-seq data for the UNC040182B tumor sample (top lane) and SUM102 cell line sample (bottom lane) for the BCL11A gene is visualized using IGV. The corresponding tiling array copy number plot for the tumor sample and gene and the associated area of genomic coverage is highlighted in the bottom panel. (PDF) [file pone.0051719.s002.pdf]

Supplementary Figure 4

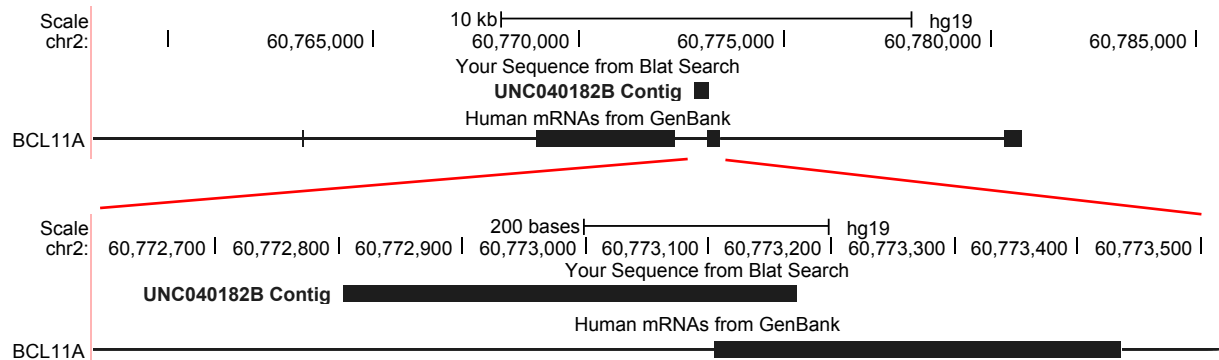

Supplement: Figure S4 — de novo assembly of targeted BCL11A micro-deletion mRNA-seq data in the UNC040182B tumor sample. The contig aligned to the region of BCL11A micro-deletion is visualized in space using the UCSC genome browser, with the location of the reference exon site displayed. A magnified view of the region is also provided. (PDF) [file pone.0051719.s004.pdf]
