# Supplementary material for: Micro-Scale Genomic DNA Copy Number Aberrations as Another Means of Mutagenesis in Breast Cancer
Source: PLoS One. 2012 Dec 17;7(12):e51719. doi: 10.1371/journal.pone.0051719 (PMC3524128; doi:10.1371/journal.pone.0051719)
Supplement: Figure S3 — de novo assembly of targeted EGFR micro-amplification mRNA-seq data in the 990141B tumor sample. A) The two contigs aligned to the region of EGFR micro-amplification are visualized in space using the UCSC genome browser. B) The aligned region of the contigs are displayed in red with the unaligned base pairs at the start and end of the sequence written out. Sites within the aligned region where the unaligned GACCT sequence are observed are highlighted with black boxes. (PDF) [file pone.0051719.s003.pdf]

Supplementary Figure 3

A)

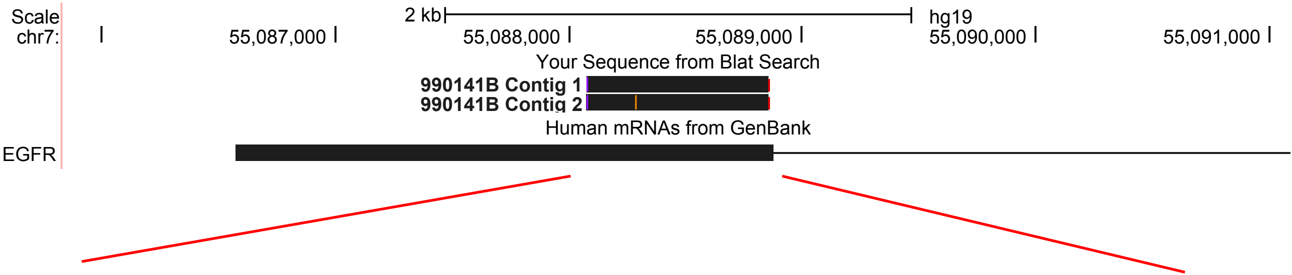

B)

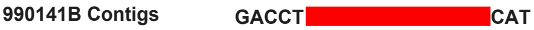

chr7:55088001-55088901

CTCCACCTGCAGCCCTTCGGTCGCGCCTGGGCTTCGCGGTGGAGCGGGACGCGGCTGTCCGGCCACTGCAGGGGGGGATCGCGGGACTCTTGAGCGGAA
GCCCCGGAAGCAGAGCTCATCCTGGCCAACACCATGGTGTTTCAAAATGGGGCTCACAGCAAACTTCTCCTCAAAACCCGGAGACTTTCTTTCTTGGATGT
CTCTTTTTGCTGTTTGAAGAATTTGAGCCAACCAAAATATTAAACCTGTCTTACACACACACACACACACACACACACACACACCGGATTGCTGTCCCTG
GTTCAAGTGTGCCAAGTGTGCAGACAGAACATGAGCGAGTCTGGCTTCGTGACTACCGACCATAAACCCACTTGACAGGGGAAACATGCCTTGGAAGGTTT
AATTGCACAATTCCAACCTTGAGCTGCGCGGGTTCCAAGAGCCAGGCCCGTACTTGCTGTTGATGTCATTGGCTTGGGGAGTTGGGGTTTGGTGCCCAGCG
CGGTCGTTGGGGGAGGGGCAAGGCATAGAACAGTGGTTCCCAGACCTTTGCTGCACATTGGAATTACCTGGGATTAAAAAAAAAAAAAATCAAAACAAAAAC
CAGTGTCTGGCTCCCGCCCCCAGACATTCTGATTTAATTGGCATGGGGCAAGACCTGGACTTGGGATTTTTTTTAATGCTCTTCATGTGATCTGTTGGGCAGC
CAGATTTGGGGATCACTAGACGGAAGAAGGATTGTTAAAGTCTCCGGAGATGTTACTTGCCAATGCTAAGAGCTCTTTGAGGACATCTGGAATTGTTACAAT
ATTGCCAAATATAGGAAAGAGGGAAAAGGTAGAGTGTGATTCCAATAATAAAGGATTCCGCTTTTCATTGAAGGAACTGGTGGAAAGGTTT
